# Recent Advances and Clinical Outcomes of Endometriosis

**DOI:** 10.3390/jcm14030798

**Published:** 2025-01-26

**Authors:** Harald Krentel, Rudy Leon De Wilde, Maya Sophie de Wilde

**Affiliations:** 1 Department of Obstetrics, Gynecology and Gynecological Oncology, Bethesda Hospital Duisburg, Heerstrasse 219, 47053 Duisburg, Germany; 2 Clinic of Gynecology, Obstetrics and Gynecological Oncology, University Hospital for Gynecology, Pius-Hospital Oldenburg, University Medicine Oldenburg, 26121 Oldenburg, Germany; rudy-leon.dewilde@pius-hospital.de (R.L.D.W.); mayadewilde@yahoo.com (M.S.d.W.)

Do recent advances positively influence clinical outcomes? Are we able to transform scientific results into daily clinical practice? Do patients have a faster and more precise diagnosis, and thus an individualized treatment approach, considering the medical, reproductive and surgical aspects? Without doubt, the current advances in endometriosis care represent a dramatic change in how patients with endometriosis should ideally be treated. A recent international intersociety consensus highlighted the role of imaging in deep endometriosis, presenting 20 statements on ultrasound, MR imaging, and classification systems [1]. Transvaginal ultrasound [2] and MR imaging [3] are non-invasive diagnostic tools that allow for a precise description of endometriosis lesions; thus, the treatment approach can be tailored to an individual’s symptoms, age, and family planning. This change from diagnostic laparoscopy to imaging-based diagnosis of the disease has also been considered in the international guidelines [4]. However, the specificity and sensitivity of both diagnostic methods depend on the individual skills and experience of the examiner [5]. Training concepts and globally accepted curricula are needed, ideally using an intersociety approach, in order to broaden the application of the cutting-edge options in diagnosing endometriosis and adenomyosis.

Whether medical, surgical, or reproductive treatment should be the first choice and the optimal moment for each type of treatment should be determined by a multidisciplinary team (MDT) [6]. New oral GnRH antagonists add to the medical treatment options in patients with symptomatic endometriosis and adenomyosis [7,8]. Their ability to control symptoms can be used to avoid surgery or postpone surgery to the optimal moment in the patient’s life [9]. According to the presurgical staging results, the surgical approach, technique, the duration of surgery, instrumentation, the possible use of multidisciplinary approaches, and the necessary preparation of the patient, including obtaining detailed informed consent, can be determined, and less invasive methods for the treatment of deep endometriosis and adenomyosis, such as high-intensity focused ultrasound (HIFU), can be considered [10,11]. However, there are still many open questions in the future of endometriosis surgery. The best approach to ovarian endometriosis is not yet clear. The impact of deep endometriosis surgery on infertility compared to reproductive treatment alone is still under debate. Although global recommendations for adhesion prevention in endometriosis surgery were recently published, the optimal strategy requires further research [12]. Does robotic-assisted surgery (RAS) offer significant advantages in complex endometriosis surgery? What is the role of simultaneous adenomyosis? The contributors to this Special Issue aimed to provide answers to some of these questions and open the door to the inclusion of new technologies, such as artificial intelligence and biomarkers. AI might make a big difference in diagnosing endometriosis and adenomyosis in the future, providing a high-precision description of all lesions in ultrasound and MR imaging and allowing for classification using all available systems simultaneously in the blink of an eye. The inclusion of these results in AI-guided RAS might take this surgical approach to the next level, avoiding individual mistakes in overlay techniques and activating warning systems before a complication occurs. Modern biomarkers, and gen-sequencing may allow for the very early diagnosis of the disease, even before the first symptoms occur [13]. MicroRNA-based tests may only be the beginning of what will become possible in the coming years [14,15]. It seems possible that the development of new drugs will make surgery unnecessary and women with endometriosis will not have to go through years of symptoms, underdiagnosis, and complex surgeries. However, there is a long way to go until this utopia becomes reality. Meanwhile, the recent advances should become the clinical standard for all women suffering from endometriosis and adenomyosis. Every single scientific work on this topic, including this Special Issue, contributes to the bigger picture in changing endometriosis care.
